# The relationship between pepsinogen C and gastric carcinogenesis: a transgene and population study

**DOI:** 10.1186/s12885-023-11020-z

**Published:** 2023-06-08

**Authors:** Ying E., Qian Yu, Tao Sun, Hang Xue, Xue-rong Zhao, Hua-chuan Zheng

**Affiliations:** 1 Department of Oncology and Central Laboratory, The Affiliated Hospital of Chengde Medical University, Chengde, 067000 China; 2 Department of Thoracic Surgery, The Affiliated Fourth Hospital of China Medical University, Shenyang, 110032 China; 3 Department of Oncology, Liaoning Cancer Hospital, Shenyang, 110042 China; 4 Department of Immunology, Basic Medicine College of Chengde Medical University, Chengde, 067000 China

**Keywords:** Transgenic mouse, Pepsinogen C, PTEN, Gastric cancer, Breast cancer, Chief cell

## Abstract

**Background:**

Pepsinogen C (PGC) is expressed in chief cells, fundic mucous neck cells, and pyloric gland cells of gastric epithelium and also in breast, prostate, lung, and seminal vesicles.

**Methods:**

We explored the clinicopathological and prognostic significances of PGC mRNA using pathological and bioinformatics analyses. We generated PGC knockout and PGC-cre transgenic mice to observe the effects of PGC deletion and PTEN abrogation in PGC-positive cells on gastric carcinogenesis. Finally, we observed the effects of altered PGC expression on aggressive phenotypes by CCK8, Annexin V staining, wound healing and transwell assays and analyzed the partner proteins of PGC using co-IP (co-immunoprecipitation) and double fluorescence staining.

**Results:**

PGC mRNA level was inversely correlated with the T and G stage and a short survival of gastric cancer (*p* < 0.05). PGC protein expression was negatively linked to lymph node metastasis, dedifferentiation, and low Her-2 expression of gastric cancer (*p* < 0.05). No difference in body weight or length was evident between wild-type (WT) and PGC knockout (KO) mice (*p* > 0.05), but *PGC* KO mice had a shorter survival than WT mice (*p* < 0.05). No gastric lesions were observed in the mucosa of the granular stomach in *PGC* KO mice, which displayed lower frequency and severity of gastric lesion than in WT mice after treated with MNU. Transgenic PGC-cre mice showed high cre expression and activity in the lung, stomach, kidney, and breast. Gastric cancer and triple-negative lobular breast adenocarcinoma were found in PGC-cre/PTEN^f/f^ mice with two previous pregnancies and breast feeding, but breast cancer was not seen in transgenic mice exposed to either estrogen or progesterone, or those with two previous pregnancies and no breast feeding. PGC suppressed proliferation, migration, invasion, and induced apoptosis, and interacted with CCNT1, CNDP2 and CTSB.

**Conclusion:**

PGC downregulation was seen in gastric cancer, but *PGC* deletion resulted in resistance to chemically-induced gastric carcinogenesis. PGC expression suppressed the proliferation and invasion of gastric cancer cells possibly by interacting with CCNT1, CNDP2 and CTSB. Spontaneous triple-negative lobular adenocarcinoma and gastric cancer were seen in PGC-cre/PTEN^f/f^ mice, and the breast carcinogenesis was closely linked to pregnancy and breast feeding, but not to single exposure to estrogen or progesterone, or pregnancy. Limiting either pregnancy or breast feeding might help to prevent hereditary breast cancer.

**Supplementary Information:**

The online version contains supplementary material available at 10.1186/s12885-023-11020-z.

## Introduction

Anatomically, the distal to proximal stomach of the mouse contains the antrum, glandular corpus, and squamous forestomach. Histologically, parietal, pit, chief, stem, mucous neck, and enteroendocrine cells are found in the gastric unit of the corpus and body, whereas only mucus-producing pit and neck cells are found in the distal antrum and mucus-producing pit cells in the upper epithelium. In particular, chief cells originate from mucous neck cells of the glandular midportion and are responsible for the release of pepsinogen, lipases, chymosin, and leptin. Under acidic conditions, pepsinogen is proteolyzed into the protease pepsin [1].

Pepsinogen is composed of chymosinogen, pepsinogen F, pepsinogen C (PGC), pepsinogen B, and pepsinogen A. In particular, PGC is an aspartic endoprotease that is secreted from chief cells as zymogen and ultimately activated as pepsin C at pH < 5. PGC is expressed in gastric chief cells, fundic mucous neck cells, pyloric glandular cells, and Brunner’s glandular cells and in extra-gastric organs, including breast, prostate, lung, and seminal vesicles. The in situ expression of PGC in gastric mucosa gradually decreases from superficial gastritis, atrophic gastritis, intestinal metaplasia, and dysplasia to gastric cancer, as found by Correa et al. [2]. Although homozygous and heterozygous deletion, genetic polymorphism, and promoter methylation result in the downregulation and loss of PGC expression, PGC overexpression might be caused by hepatocyte growth factor and hormonal (androgens, glucocorticoids, and progesterone) stimuli [3]. PGC expression levels were apparently up-regulated in hepatocellular carcinoma, prostate cancer, breast cancer, ovarian cancer, endometrial cancer, pancreatic cancer, kidney cancer, bladder cancer, eyelid basal cell carcinoma, squamous cell carcinoma and melanoma. PGC was a valuable prognostic factor that indicated better prognosis and longer survival of prostate cancer, breast cancer and ovarian cancer [3].

The promoters of the β- subunit of H ( +)-, K ( +)-ATPase gene (Atp4b) [1], cytokeratin 19 [4], and Lgr5 [5] have been used to drive cre expression in parietal cells, stem-like cells, and gastric progenitors. Oshima et al. [4] established a gastric tumor model using K19-Wnt1/C2mE mice. Atp4b promoter has been used to guide SV40 T antigen expression to generate a transgenic mouse model of metastatic gastric cancer [6] and synergistically drive both E-cadherin and p53 knockout (KO) for a metastatic diffuse-type gastric cancer model [7]. By mediating Smad4 and PTEN deletion, Lgr5 promoter leads to invasive intestinal-type gastric cancer [5]. However, there has been no report on the effects of either PGC knockout or genetic alterations in chief cells on the phenotypes of transgenic mouse models. Thus, to study the relationship between PGC loss and gastric carcinogenesis, we generated PGC KO mice and detected the presence of gastric lesions, with or without treatment with the chemical carcinogen *N*-nitroso-*N*-methylurea (MNU). In addition, we generated PGC-cre mice to conditionally knock out the tumor suppressor gene PTEN (phosphatase and tensin homolog) to determine the effects of the genetic alteration on gastric carcinogenesis and histogenesis.

## Materials and methods

### Subjects

Gastric cancer tissues (*n* = 312) were sampled from The First Affiliated Hospital of Jinzhou Medical University. They were routinely prepared for pathological blocks and then for tissue microarray. The patients’ average age at operation was 57 years (range, 27–85 years). There were 221 cases of lymph node metastasis and 15 cases of distant metastasis. In addition, we also retrieved 3166 cases of breast cancer from Liaoning Cancer Hospital and collected their pregnancy information and molecule subtyping. These patients had not received any adjuvant treatment, radiotherapy, or chemotherapy before the operation. They signed informed consent, and the ethics committees of The Affiliated Hospital of Chengde Medical University approved the study.

### Generation and care of transgene mice

PGC KO and PGC-cre transgenic mice were generated using the CRISPR/Cas9 system in Shanghai Biomodel Organism Science & Technology Development Co. Ltd. To check cre activity, we crossed B6/JGpt-H11em1Cin (CAG-loxp-ZsGreen-stop-loxp-tdTomato)/Gpt, and PGC-cre mice with B6.129S4-PTEN^tm1^Hwu/J (Jax Lab) mice. To chemically induce gastric neoplasia, we orally administered MNU (240 mg/L) to PGC KO mice at 1-week intervals from the 8^th^ and 38^th^ week for 10 weeks. Moreover, we exposed virgin 6-to-8-week-old PGC-cre/PTEN^f/f^ mice (*n* = 5/group) to 17β-estradiol (0.18 mg/90-day release × 2; Innovative Research of America) or progesterone (1 mg/week for 3 continuous months). The mice were kept in a specific pathogen-free (SPF) and temperature-controlled facility with a 12-h light/dark cycle and fed with rodent food for pregnant mice (Beijing HFK Bioscience) and autoclaved water. All procedures were approved by the Committee for Animal Welfare and Management of The Affiliated Hospital of Chengde Medical University.

### Cell culture and transfection

Gastric cancer (AGS, GT-3, HGC-27, Kato-III and SCH) cell and normal epithelial (GES-1) cells come from Cell bank of Chinese Academy of Sciences, Shanghai, China. They were maintained in RPMI 1640 or DMEM medium supplemented with 10% fetal bovine serum (FBS), 100 units/mL penicillin, and 100 μg/mL streptomycin, in a humidified atmosphere of 5% CO_2_ at 37 °C. Plasmid pHG-CMV-Kan2-PGC (Honorgen) was used to ectopically overexpress PGC in AGS cells, and PGC siRNA to knockdown PGC in GES-1 cells. We transfected plasmid and siRNA (General BioL) using Lipofectamine 3000 (Thermo Fisher Scientific). The siRNA target sequences were sense: 5’-CGUGAGACCAUGAAGGAGATT-3’ and antisense: 5’-UCUCCUUCAUGGUCUCACGTT-3’ for PGC siRNA1, sense: 5’-CCUAC GAGCCCAUGGCCUATT-3’ and antisense: 5’-UAGGCCAUGGGCUCGUAGGTT-3’ for PGC siRNA2, sense: 5’-GAGUUCGGCUUGAGUGAGATT-3’ and antisense: 5’-UCUCACU CAAGCCGAACUCTT-3’ for PGC siRNA3, sense: 5’-UUCUCCGAACGUGUCACGUTT-3’ and antisense: 5’-ACGUGACACGUUCGGAGAATT-3″ for mock, and sense: 5’-GUAUGAC AACAGCCUCAAGTT-3’ and antisense: 5’-CUUGAGGCUGUUGUCAUACTT-3’ for GAPDH as negative control.

### Proliferation assay

The Cell Counting Kit-8 was used to count the number of viable cells. 2.0 × 10^3^ cells per well were planted on a 96-well plate and cultured until adherence. At multiple consecutive points in time, 10 μL of CCK-8 solution was added to each well of the plate, and the plates were incubated for 2 h until being measured at 450 nm.

### Apoptosis assay by flow cytometry

Flow cytometry was performed with Fluorescein isothiocyanate isomer (FITC)-labeled Annexin V (BD Pharmingen, USA) and Phycoerythrin (PE). Among them, FITC-labeled Annexin V was detected for phosphatidylserine externalization as an endpoint indicator of early apoptosis recommended by the protocol. And PE was used to detect late apoptosis and necrosis.

### Wound healing assay

In 6-well culture plates, cells were seeded at a density of 1.0 × 10^6^ cells/well. After reaching confluence, the cell monolayer was scraped with a pipette tip to form a scratch, washed three times with PBS, and cultured in FBS-free medium. The cells were pictured at 0 h and 72 h of incubation and the scratch area was calculated using Image J software.

### Transwell assay

In the migration assay, 2.5 × 10^5^ cells were resuspended in serum-free RPMI 1640 or DMEM medium, and planted in the upper portion of the uncoated chamber (BD Bioscience). The lower part of the chamber contained 10% FBS as a chemoattractant. After 24 h in the incubator, the cells were wiped on the membrane, cleaned with PBS, fixed in 100% methanol, and stained with crystalline violet. In the invasive assay, the insert membranes were coated with diluted matrigel (BD Bioscience), and the other procedures were consistent with above-mentioned.

### PCR

We used the standard phenol/chloroform approach to isolate DNA from the tail and various organs of mice. PCR was used to identify the genetic phenotype using tail DNA as template and by targeting PGC, cre, and PTEN with Takara polymerase. The primers were as follows: forward, 5'-GTTTGAGCGAGGGAGGAA-3', and backward, 5'-GCCAGGGTCATACTTGT G-3' (336 bp) for wild-type (WT) PGC; forward, 5'-GGGGTGAGGATGGATAAA-3', and backward, 5'-TGAGTGTAGTAGGTGGAGGA C-3' (748 bp) for PGC KO; forward, 5'-GCCT GCATTACCGGTCGATGC-3', and backward, 5'-CAGGGTGTTATAAGCAATCCC-3' for cre (481 bp); forward, 5'-CAAGCACTCTGCGAACTGAG-3', and backward, 5'-AAGTTTTT GAAGGCAAGATG C-3' for PTEN (WT, 156 bp; ∆5, 328 bp). To verify PTEN KO (knockout) in target organs, we performed PCR targeting PTEN with the reported primers [8]. The primers for report gene mice were H11-wt-tF1, 5’-CAGCAAAACCTGGCTGTGGAT C-3’; H11-wt- tR1, 5’-ATGAGCCACCATGTGGGTGTC-3’ for wt B6/JGpt-H11em1Cin (CAG-LoxP- ZsGreen-Stop-LoxP-tdTomato)/Gpt (412b); H11-PolyA- 3tF2, 5’-CCTCCTCTCCTGACTAC TCCCAGTC-3’ and H11-tR2, 5’-TCACAGAAACCATATGGCGCTCC-3’ for PGC-cre/B6/ JGpt- H11em1Cin (CAG-LoxP-ZsGreen-Stop-LoxP- tdTomato)/Gpt (1229 bp).

### RT-PCR

We extracted RNA from various organs of the transgene mice, or gastric cancer or epithelial cells and synthesized cDNA using transcriptase and hexamer primer. SYBR Premix Ex Taq™ II kit (Takara, Japan) was used to amplify the cre gene (using the above-mentioned primers) with mouse GAPDH as an internal control (forward, 5'-CAACGACCCCTTCATTGACC-3'; backward, 5'-GGCTTCCCGTTGATGACAAG-3'). To verify the PGC expression, we used Hotstart polymerase (Takara) to amplify mouse PGC (forward, 5'-ATGAAGTGGATGGTGG TCGC-3'; backward, 5'-GGAAGTTCTGGGGTGGAGTC-3') with GAPDH as control and performed the electrophoresis on a 1% agarose gel. To observe the efficacy of PGC overexpression or knockdown, we employed amplified human PGC with GAPDH as internal control by real-time RT-PCR. Human GAPDH primers were forward: 5’-GTCTCCTCTGACT TCAACAGCG-3’ and backward: 5’-ACCACCCTGTTGCTGTAGCCAA-3’. Human PGC primers were forward: 5'⁃CCAGGAGTTCGGCTTGAGT⁃3' and reverse: 5'⁃CCACGGACAG AGCAGGGTAG-3 '. Gene expression was calculated as 2^−∆Ct^, where ∆Ct was equal to Ct (gene) − Ct (GAPDH).

### Co-immunoprecipitation (Co-IP)

Co-IP was carried out to check the patterner protein of PGC. Briefly, at least 1 mg protein was pre-cleared with 50 μl protein A-Sepharose beads for 1 h, and incubated with 5 μg mouse anti-PGC antibody (Santa Cruz). We removed non-specific binding proteins by washing and centrifugation. Finally, protein was sampled by heating in 50 μl 2 × SDS sample buffer.

### Western blot

We homogenized mouse tissues in RIPA lysis buffer to isolate the protein and performed a protein assay. Denatured protein was separated in SDS-PAGE and electrically transferred onto PVDF membrane. The membrane was blocked with 5% milk, incubated with rabbit anti-cre (Novus, USA), mouse anti-PGC (Santa Cruz), mouse anti-GAPDH (Proteintech), mouse anti-Akt (Proteintech), rabbit anti-APOA1 (Proteintech), rabbit anti-β-catenin (Proteintech), rabbit anti-CCNT1 (Proteintech), rabbit anti-CNDP2 (Proteintech), rabbit anti-CTSB (Proteintech), rabbit anti-E-cadherin (Proteintech), mouse anti-EGFR (Proteintech), rabbit anti-FGG (Proteintech), rabbit anti-N-cadherin (Proteintech), and rabbit anti-PTEN (Proteintech). After wash three times by TBST, the membrane was then incubated with HRP-labeled secondary antibody (DAKO). Finally, protein-specific antibody binding was visualized using an ECL luminescence solution.

### Double staining

To clarify the colocalization of PGC and its partner expression in gastric cancer, we performed double fluorescent immunostaining. The anti–mouse PGC and anti-rabbit CCNT1, CNDP2 or CTSB antibodies were mixed and incubated with AGS and its PGC transfectant cells. Both Alexa Fluor 488 (green) donkey anti-rabbit and Alexa Fluor 568 (red) anti-mouse IgG (Invitrogen, USA; 1:500) were used as secondary antibodies. DAPI was used to stain the nuclei.

### Pathological examination

Mouse tissues were fixed in 20% formalin in PBS and embedded in paraffin to prepare blocks according to the standard protocol. The blocks were sectioned into 4-μm-thick slides for hematoxylin–eosin staining. For immunohistochemistry, we deparaffinized and dehydrated these slides and then performed antigen retrieval. We blocked endogenous HRP by using 3% H_2_O_2_ and non-specific binding by using 5% BSA. Next, we incubated the slides with the anti-PGC (Invitrogen), anti-cre (Novus), anti-PTEN (Cell Signaling), anti-CA153 (Abcam), anti-GATA3 (Abcam), anti-estrogen receptor (ER) (Abcam), anti-progesterone receptor (PR) (Abcam), anti-c-erbB-2 (Abcam), or anti-Ki67 (Abcam) antibody for 1 h and then with Envison-PO secondary antibody for 1 h. All sections were colored with DAB and counterstained with Mayer’s hematoxylin. We omitted the primary antibody as a negative control.

### Report gene assay

The stomach, kidney, lung and breast tissues of the PGC-cre/B6/JGpt-H11em1Cin (CAG-loxp-ZsGreen-stop-loxp-tdTomato)/Gpt and B6/JGpt-H11em1Cin(CAG-loxp- ZsGreen- stop-loxp-tdTomato)/Gpt mice were removed and were embedded into OCT compound. The blocks were sectioned at 4 µm, and e fixed in 4% paraformaldehyde at room temperature. The slides were stained by DAPI and subjected to the observation under the fluorescence microscopy. After activation, the green color become red in the target cells after the activation using cre recombinase.

### Serum measurement

The peripheral blood of nude mice was collected from abdominal vein after being killed by cervical dislocation, kept into a venous blood sample collection vessel, and centrifuged at 4000 rpm per minutes for 5 min. The peripheral blood was routinely examined by MS9 automatic hematology analyzer (France), and the supernatant was analyzed for hepatic and renal function examination by automatic biochemical analyzer (Hitachi 7600, Japan). Plasma samples were deproteinized with 5% trichloroacetic acid solution and centrifuged at 10,000 rpm for 15 min at 4 °C before measurement. The supernatant fractions were filtered through regenerated cellulose (Millipore, Bedford, MA, USA). The amino acid concentrations in the plasma were measured by HPLC-electrospray ionization-mass spectrometry followed by derivatization as described previously [9].

### Bioinformatics analysis

The mRNA expression of PGC was analyzed using the xiantao platform (http://www.xiantao.love) and UALCAN (http://ualcan.path.uab.edu/). We compared the mRNA expression of PGC with the clinicopathological characteristics of patients with gastric cancer using TCGA database. Their prognostic significance was analyzed using Kaplan–Meier plotter (https://www.kmplot.com/). BioGRID (https://thebiogrid.org/111246), IntAct (https://www.ebi.ac.uk/intact/search?query=id:P20142*#interactor) and STRING (https://string-db.org/network/9606.ENSP00000362116) databases were used to predict the interacting partners of PGC.

### Statistical analysis

SPSS version 26.0 software was used to statistically analyze the data. A Spearman correlation test was performed for rank data, a student’s t test for mean comparisons, and a chi-square test for rate comparisons. The Kaplan–Meier method was used to analyze univariate survival data. A *p*-value < 0.05 was considered statistically significant.

## Results

### Relationship between PGC and the clinicopathological features of gastric and breast cancer

**Fig. 1:**
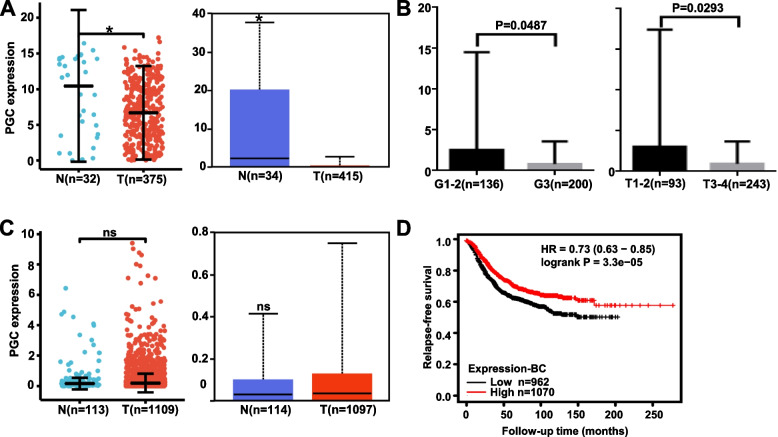
Clinicopathological and prognostic significance of PGC mRNA expression in either gastric or breast cancer. PGC mRNA expression was downregulated in gastric cancer according to xiantao and UALCAN databases (**A**). TCGA data were used to compare PGC mRNA expression with the clinicopathological features of gastric cancer (**B**). There was no difference in PGC mRNA expression between breast cancer and normal tissues using xiantao and UALCAN databases (**C**). According to a Kaplan–Meier plot, PGC mRNA expression was positively related to the relapse-free survival rate of patients with breast cancer (**D**). Note: N, normal tissue; T, tumor tissue; ns, not significant; HR, hazard ratio

We found lower mRNA expression of PGC in gastric cancer than in normal mucosa in both the xiantao and UALCAN databases (Fig. 1A, both *p* < 0.05). According to TCGA, PGC mRNA expression was inversely correlated with the T and G stage of gastric cancers (Fig. 1B, *p* < 0.05). Immunohistochemically, PGC protein expression was negatively associated with dedifferentiation, lymph node metastasis, and low Her-2 expression of gastric cancer (; Table 1, *p* < 0.05). The Kaplan–Meier plot indicated a positive relationship between PGC mRNA expression and overall survival in gastric cancer patients with T2, T3, N1 + 2 + 3, N1, N2, N3, M0, or IV stage, diffuse subtype, or Her-2-positive disease (*p* < 0.05, data not shown). The progression-free survival was longer in PGC-positive patients than in PGC-negative patients in the female gastric cancer group, the T3, N1 + 2 + 3, N2, M0, or IV stage groups, those with the poorly differentiated subtype, and those treated with 5-FU adjuvant (*p* < 0.05, data not shown). The same pattern was seen for post-progression survival in gastric cancer patients with T3, N1 + 2 + 3, or III stage or those treated with surgery alone (*p* < 0.05, data not shown).

**Table 1:** The relationship between PGC protein expression and the clinicopathological characteristics of gastric cancer

Clinicopathological features	n	PGC expression				PR (%)	*P* value
		**-**	** + **	** + + **	** + + + **		
Gender							0.889
Male	229	159	58	11	1	30.6	
Female	83	58	22	3	0	30.1	
Age(years)							0.471
< 65	195	133	54	7	1	31.8	
≥ 65	117	84	26	7	0	28.2	
Gross classification							0.743
Ulcerative	274	188	72	13	1	31.4	
Infiltrative	11	10	1	0	0	9.1	
Protrusive	14	11	3	0	0	21.4	
Tumor size (cm)							0.307
< 4	121	83	29	8	1	31.4	
≥ 4	184	128	50	6	0	30.4	
Depth of invasion							0.338
Tis-T2	72	50	18	3	1	30.6	
T3-T4	240	167	62	11	0	30.4	
Lymphatic invasion							0.779
No	171	119	44	7	1	30.4	
Yes	128	87	34	7	0	32.0	
Lymph node metastasis							0.026
No	89	55	25	8	1	38.2	
Yes	221	160	55	6	0	27.6	
Distant metastasis							0.970
No	297	207	76	13	1	30.3	
Yes	15	10	4	1	0	33.3	
Differentiation							< 0.001
Poorly	214	160	48	6	0	25.2	
Well & moderately	78	40	29	8	1	48.7	
Lauren’s classification							0.003
Intestinal-type	120	71	40	8	1	40.8	
Diffuse-type	144	117	25	2	0	18.7	
Mixed-type	48	29	15	4	0	39.6	
HER2 expression							0.037
Low (- ~ +)	63	44	17	2	0	30.2	
High (+ + ~ + + +)	20	9	7	3	1	36.1	

*PR *Positive rate

There was no difference in PGC mRNA expression between breast cancer and normal tissue according to the xiantao and UALCAN databases (Fig. 1C, *p* > 0.05). As summarized in Table 2, PGC mRNA expression was higher in invasive lobular carcinoma than in ductal carcinoma (*p* < 0.05), positively associated with ER and PR expression in breast cancer (*p* < 0.05), and negatively associated with the aggressiveness of PAM50 subtypes (*p* < 0.05). PGC mRNA expression was positively linked to the relapse-free survival rate of the breast cancer patients (Fig. 1D, *p* < 0.05).

**Table 2:** The relationship between PGC mRNA expression and the clinicopathological characteristics of breast cancer

Clinicopathological features	Variable	Low expression	High expression	P
Age, n (%)	≦60	292 (27%)	309 (28.5%)	0.345
	> 60	249 (23%)	233 (21.5%)	
T stage, n (%)	T1	137 (12.7%)	140 (13%)	0.537
	T2	321 (29.7%)	308 (28.5%)	
	T3	62 (5.7%)	77 (7.1%)	
	T4	19 (1.8%)	16 (1.5%)	
N stage, n (%)	N0	259 (24.3%)	255 (24%)	0.136
	N1	177 (16.6%)	181 (17%)	
	N2	64 (6%)	52 (4.9%)	
	N3	29 (2.7%)	47 (4.4%)	
M stage, n (%)	M0	456 (49.5%)	446 (48.4%)	0.111
	M1	6 (0.7%)	14 (1.5%)	
Pathologic stage, n (%)	Stage I	91 (8.6%)	90 (8.5%)	0.304
	Stage II	311 (29.3%)	308 (29.1%)	
	Stage III	118 (11.1%)	124 (11.7%)	
	Stage IV	5 (0.5%)	13 (1.2%)	
Histological type, n (%)	IDC	417 (42.7%)	355 (36.3%)	< 0.001
	ILC	71 (7.3%)	134 (13.7%)	
PR status, n (%)	Negative	200 (19.3%)	142 (13.7%)	< 0.001
	Indeterminate	2 (0.2%)	2 (0.2%)	
	Positive	313 (30.3%)	375 (36.3%)	
ER status, n (%)	Negative	146 (14.1%)	94 (9.1%)	< 0.001
	Indeterminate	2 (0.2%)	0 (0%)	
	Positive	367 (35.5%)	426 (41.2%)	
HER2 status, n (%)	Negative	286 (39.3%)	272 (37.4%)	0.843
	Indeterminate	7 (1%)	5 (0.7%)	
	Positive	83 (11.4%)	74 (10.2%)	
PAM50, n (%)	Normal	16 (1.5%)	24 (2.2%)	< 0.001
	Luminal A	244 (22.5%)	318 (29.4%)	
	Luminal B	108 (10%)	96 (8.9%)	
	Her2 +	32 (3%)	50 (4.6%)	
	Basal	141 (13%)	54 (5%)	

*ILC* Invasive lobular carcinoma, *IDC* Invasive ductal carcinoma, *ER* Estrogen receptor, *PR* Progesterone receptor

### Effects of PGC expression on physiology and gastric carcinogenesis in PGC KO mice

**Fig. 2:**
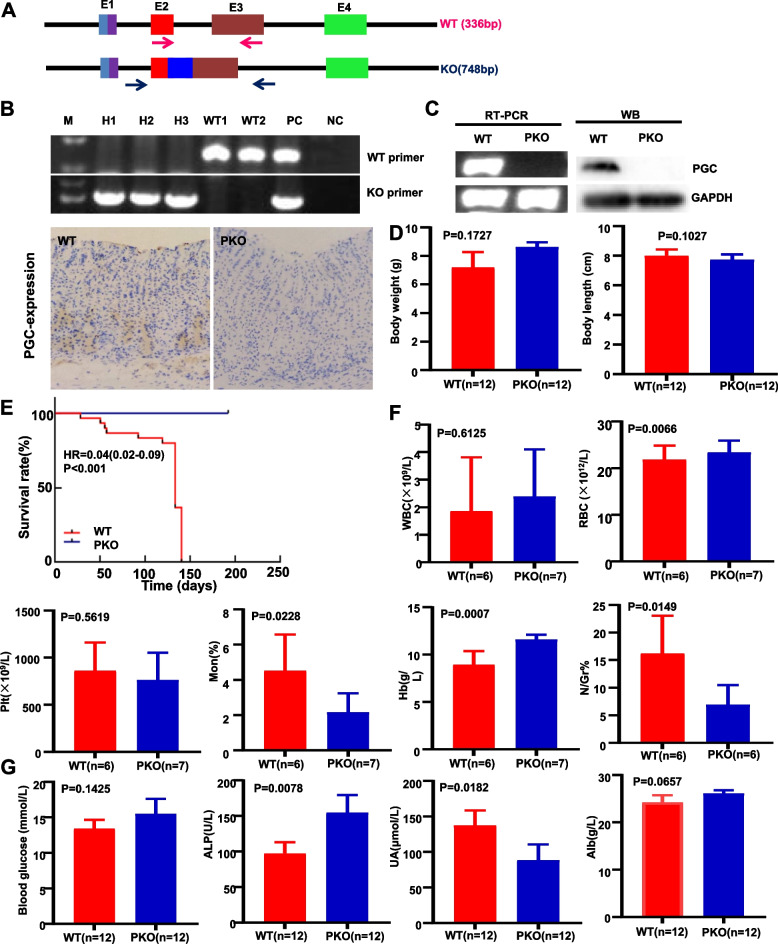
Effects of PGC KO on the phenotypes of transgene mice. PGC knockout (PKO) mice were generated according to the schematic diagram using the CRISPR/Cas9 technique (**A**). Wild-type (WT) and homozygous (H) PKO mice were genetically identified by PCR of mouse tail DNA (**B**). PGC expression loss was seen in PKO mice compared with WT mice according to RT-PCR, western blot, and immunohistochemistry (**C**). Body weight and length were compared between WT and PKO mice (**D**). Survival curves of these three types of mice were plotted using the Kaplan-Meier method (**E**). Routine blood test (**F**) and hepatic and renal function tests (**G**) were compared between the WT and PKO mice. Note: E, exon; M, marker; NC, negative control; PC, positive control; RT-PCR, reverse transcription- polymerase chain reaction; WB, western blot; WBC, white blood cell; RBC, red blood cell; Plt, platelet; Mon, monocyte; Hb, hemoglobin; N/Gr, neutrophil/granulocyte; ALP, alkaline phosphatase; UA, uric acid; Alb, albumin

PGC KO mice were established by merging parts of exons 2 and 3 of the PGC gene with an additional insert (Fig. 2A) and were verified using PCR of mouse tail DNA (Fig. 2B). PGC expression was evident in gastric epithelial cells in WT mice but not in PGC KO mice by RT-PCR, western blot, or immunohistochemistry (Fig. 2C). No difference in body weight or length was seen between WT and KO mice (Fig. 2D, *p* > 0.05). Survival times were shorter in PGC KO mice than in WT mice (*p* < 0.05; Fig. 2E). There were similar levels of white blood cells, platelets, blood glucose, and albumin in WT and PGC KO mice (*p* > 0.05; Fig. 2F and G). Although red blood cell, hemoglobin, and alkaline phosphatase levels were lower in WT mice than in PGC KO mice (*p* < 0.05), the opposite was seen for monocytes, neutrophils/ granulocytes, and uric acid (Fig. 2F and G, *p* < 0.05). There was no difference in the serum level of 22 amino acids between WT and KO mice (*p* > 0.05, data not shown).

**Fig. 3:**
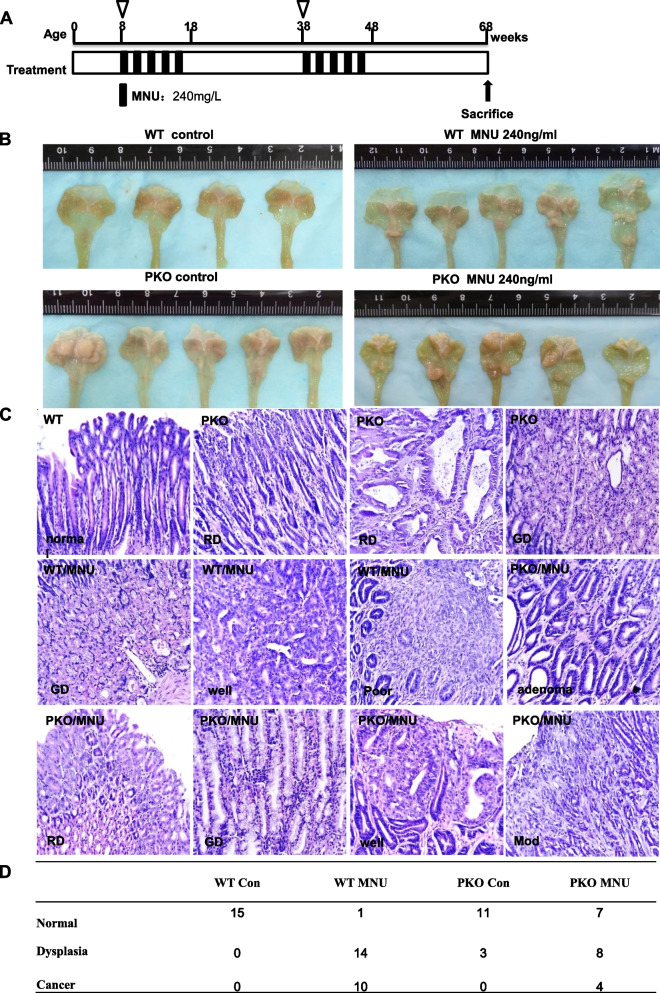
Preventive effects of homogenous PGC deletion on chemically-induced gastric carcinogenesis. MNU was orally administered to WT and PGC KO mice according to the schedule (**A**). The stomach of WT and KO mice was grossly (**B**) and histologically (**C**) observed until 68 weeks. Gastric lesions were histologically summarized (**D**). Note: WT, wild-type; PKO, PGC knockout; RD, regenerative dysplasia; GD, globoid dysplasia; Well, well-differentiated adenocarcinoma; Mod, moderately differentiated adenocarcinoma; Poor, poorly differentiated carcinoma

Grossly and histologically, no gastric lesions were observed in the mucosa of the forestomach and granular stomach of PGC KO mice (data not shown). To verify the role of PGC in gastric carcinogenesis, we orally administered MNU to PGC KO and WT mice, as illustrated (Fig. 3A), and found that the frequency and severity of gastric protrude lesions were grossly higher in WT than PGC KO mice (Fig. 3B). Histologically, normal gastric epithelium was observed in WT mice, whereas regenerative and globoid dysplasia was seen in PGC KO mice (Fig. 3C). MNU-treated WT mice exhibited globoid dysplasia and well- and poorly differentiated carcinoma, whereas MNU-treated PGC KO mice showed adenoma, regenerative and globoid dysplasia, and well- and moderately differentiated adenocarcinoma (Fig. 3C). As shown in Fig. 3D, chemically-induced lesion severity was lower in PGC KO mice than in WT mice.

### Effects of PTEN abrogation in PGC-positive cells on breast carcinogenesis

**Fig. 4:**
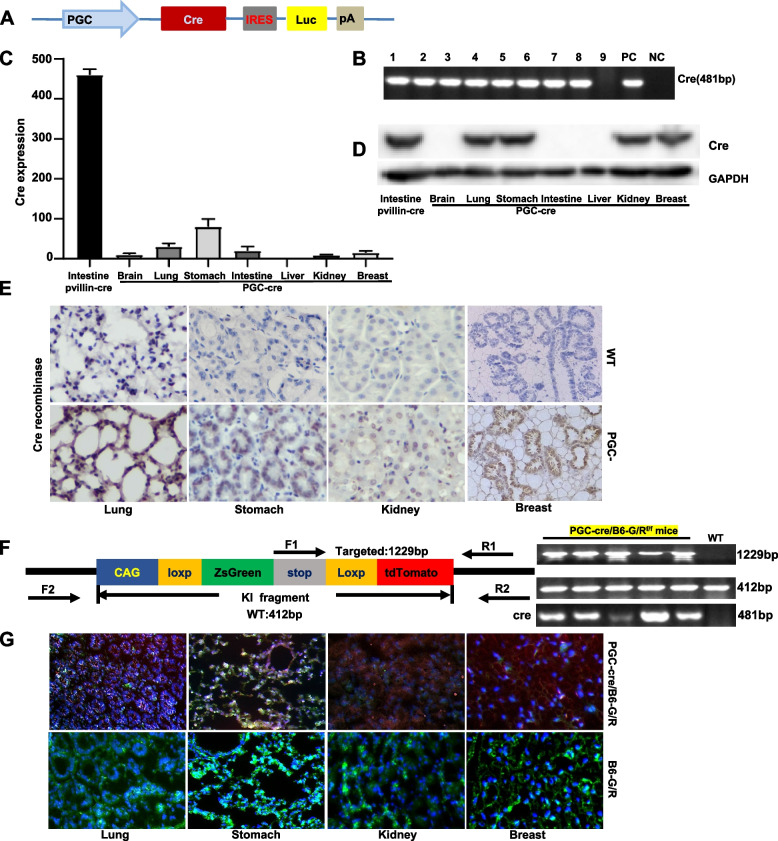
Expression and activity of cre in PGC-cre transgenic mice. Transgenic cre mice were established according to the schematic diagram using the PGC promoter (**A**). We identified nine cre-positive founders using PCR of mouse tail DNA (**B**). Cre expression levels were detected by real-time RT-PCR (**C**), western blot (**D**), and immunohistochemistry (**E**). PGC-cre/B6/JGpt-H11em1Cin (CAG-LoxP-ZsGreen-stop-Loxp- tdTomato)/Gpt (abbreviated as B6-G/R) mice were genetically screened by PCR of mouse tail DNA (**F**). The red fluorescence to indicate cre activity was seen in gastric, bronchoalveolar, kidney tubular, ductal and lobular epithelial cells of PGC-cre/B6-G/R mice, but green fluorescence was seen in the B6-G/R mice (**G**). Note: PC, positive control; NC, negative control; WT, wild-type C57 mouse

We generated a transgenic mouse in which a cre-coding region was arranged after a 2.34-kb mouse PGC promoter before an IRES, followed in order by SecNano and SV40 poly A (PGC-cre-IRES-SecNanoLuc), as shown in Fig. 4A. To develop PGC-cre transgenic mice, we microinjected linear DNA into fertilized oocytes. The tail DNA of the mice was amplified by cre primers and positive bands were seen (Fig. 4B). Cre mRNA expression was strong in the stomach, lung, intestine, breast, and kidney on quantitative RT-PCR (Fig. 4C), whereas its protein overexpression was detected in the stomach, breast, lung, and kidney (Fig. 4D). Immunohistochemically, cre expression was found in the nuclei of gastric, breast lobular, bronchial, and renal tubular epithelial cells (Fig. 4E). To confirm the cre activity, we crossed PGC-cre mice with B6/JGpt-H11em1Cin (CAG-LoxP-ZsGreen-Stop-LoxP-tdTomato)/Gpt mice, which were genetically determined by PCR of mouse tail DNA (Fig. 4F). Report red fluorescence showed that cre activity was seen in gastric, bronchioalveolar, kidney tubular, ductal and lobular epithelial cells of PGC-cre/B6/JGpt-H11em1Cin (CAG-loxp-ZsGreen-stop- loxp-tdTomato)/Gpt mice, but green fluorescence was seen in the corresponding cells of the B6/JGpt-H11em1Cin (CAG-loxp-ZsGreen-stop-loxp- tdTomato) mice (Fig. 4G).

**Fig. 5:**
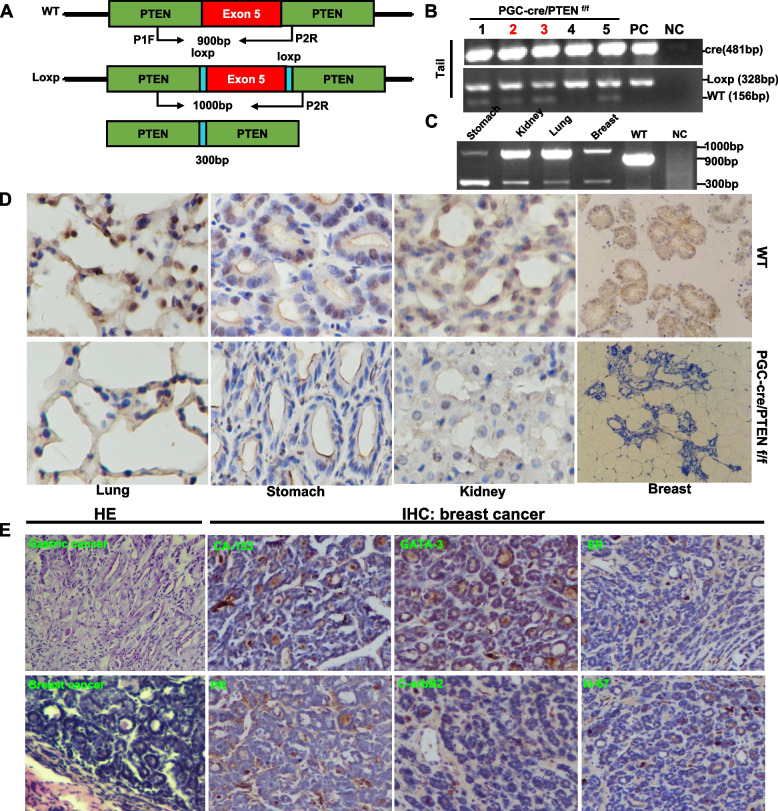
Breast carcinogenesis in transgenic mice with tissue-specific abrogation of PTEN. Primers were designed targeting the PTEN gene to identify the deletion of exon 5 (**A**). Different tissues and tail samples from PGC-cre/PTEN^f/f^ mice were subjected to DNA extraction and PCR amplification using the above-mentioned primers (**B**). PTEN deletion was also seen in the lung, stomach, kidney, and breast using PCR(**C**) and immunohistochemistry (IHC, **D**). Histologically, we observed gastric cancer and identified the breast tumor as lobular carcinoma and immunohistochemically as triple-negative breast cancer (**E**). Note: WT, wild-type; P, primer; F, forward; R, reverse; NC, negative control; HE, hematoxylin–eosin

Finally, we crossed the PGC-cre mice with PTEN^f/f^ mice (Fig. 5A and B). The model mice lacked the PTEN gene in the lung, stomach, and kidney on PCR (Fig. 5C) and immunohistochemistry (Fig. 5D). Strangely, breast cancer was grossly observed in PGC-cre/ PTEN^f/f^ mice that had more than two previous pregnancies, but not in the virgin conditional KO mice. According to histological and immunohistochemical evidence, the spontaneous breast cancer was triple-negative lobular adenocarcinoma (Fig. 5E). As shown in Supplementary table 1, the overall tumor incidence rate was 69.6% (16 of 23). Female mice had breast cancer alone, with a tumor incidence rate of 93.3% (14 of 15). Male mice had gastric cancer alone, with a tumor incidence rate of 25% (2 of 8). To establish the relationship between sexual hormones and breast carcinogenesis, we administered estrogen or progesterone to virgin PGC-cre/PTEN^f/f^ mice. Strangely, the conditional KO virgin mice did not develop breast cancer after exposure to estrogen or progesterone. So we made conditional KO mice only give birth but not breastfeeding, and even mice that gave birth more than twice did not develop breast cancer (data not shown).

### Correlation between pregnancy and breast cancer risk

In our breast cancer patients, the rate of ductal adenocarcinoma was 93.6% (2962 of 3166), whereas that of lobular adenocarcinoma was only 6.4% (204 of 3166). Breast cancer morbidity decreased as the parity number increased, as summarized in Supplementary table 2. The same results were obtained for ductal adenocarcinoma and lobular adenocarcinoma, triple-negative breast cancer (TNBC), Her-2-positive cancer, and luminal cancer. In addition, there were no differences in the correlation of pregnancy with breast cancer according to the histological and molecular subtype (*p* > 0.05). Notably, the breast cancer rate was higher in Her-2-positive cases without previous pregnancy than in the luminal and TNBC subgroups (*p* < 0.05). Of our breast cancer patients, only one patient with TNBC and lobular adenocarcinoma did not have a history of pregnancy.

### Effects of PGC expression on cellular phenotypes of PGC in gastric cancer and epithelial cells

**Fig. 6:**
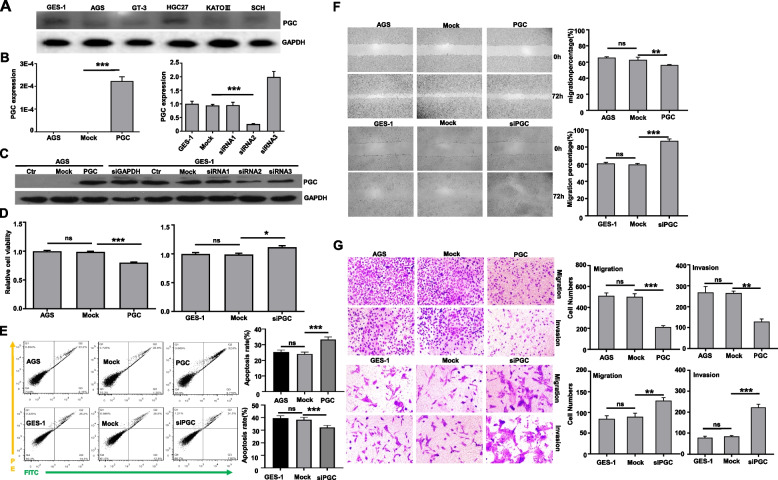
The effects of PGC on the phenotypes of gastric cancer or epithelial cells Gastric cancer and epithelial cells were subjected to the screening of PGC protein expression by Western blot (**A**). Among them, AGS was transfected with pHG-CMV-Kan2-PGC plasmid and GES-1 was transfected with siRNAs of PGC, which were verified by real-time RT-PCR (**B**) and Western blot (**C**). Among siRNAs, siRNA2 of PGC was selected for the following experiments due to significant PGC knockdown. Proliferation, chemosensitivity to 5-FU, apoptosis, migration, invasion and lipid droplet formation were determined by CCK-8 (**D**), Annexin V staining (**E**), wound healing (**F**) and transwell (**G**). Note: ns, not significant; ctr, control; *, *p* < 0.05; **, *p* < 0.01; ***, *p* < 0.001

Firstly, we screened PGC protein expression in gastric cancer or epithelial cells by Western blot (Fig. 6A), and selected AGS for PGC overexpression and GES-1 for PGC knockdown. After transfection of pHG-cmv-Kan2-PGC and PGC siRNA2, PGC became strong in AGS cell and weak in GES-1 cells according to real-time RT-PCR (Fig. 6B) and Western blot (Fig. 6C). PGC overexpression reduced the proliferation, induced apoptosis, suppressed migration and invasion by CCK-8 assay (Fig. 6D, *p* < 0.05), Annexin V staining (Fig. 6E, *p* < 0.05), would healing (Fig. 6F, *p* < 0.05) and transwell assays (Fig. 6G, *p* < 0.05). In contrast, PGC silencing had the opposite results (Fig. 6D-G, *p* < 0.05).

### The partner proteins of PGC in gastric cancer cells

**Fig. 7:**
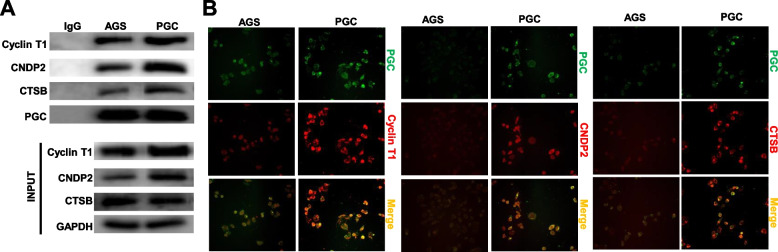
The partner proteins of PGC in gastric cancer cells. Co-IP demonstrated that PGC interacted with CCNT1, CNDP2 and CTSB in AGS cells, which became stronger in their PGC transfectants (**A**). Double fluorescence staining showed the co-localization of PGC and CCNT1, CNDP2 or CTSB in AGS cells and their transfectants (**B**)

Bioinformatics analysis showed that PGC might interact with ATP4A (ATPase H + /K + transporting subunit β), CCNT1 (cyclin T1), CDK9 (cyclin-dependent kinase 9), ESR1 (estrogen receptor 1), DHH (desert hedgehog protein), KRAS (GTPase kras), MAT2B (methionine adenosyltransferase 2 subunit β), TEX101 (testis-expressed protein 101) and WFDC8 (WAP four-disulfide core domain protein 8), CTSB (Cathepsin B), CNDP2 (Carnosine dipeptidase 2), F13B (coagulation factor XIII B chain), PGM1 (phosphoglucomutase 1), AK2 (adenylate kinase 2), MPG (N-Methylpurine DNA glycosylase), VPS18 (vacuolar protein sorting 18), PDE5A (phosphodiesterase 5A), and NPR1 (natriuretic peptide receptor 1). According to the prediction and our knowledge, we chose Akt, APOA1 (apolipoprotein A1), β-catenin, CCNT1, CNDP2, CTSB, E-cadherin, EGFR, FGG (fibrinogen γ chain), N-cadherin, and PTEN for Co-IP. As a result, Co-IP demonstrated that PGC bound to CCNT1, CNDP2 and CTSB in AGS cells, which was strengthened by PGC overexpression (Fig. 7A). Double immunofluorescence displayed the colocalization of PGC with CCNT1, CNDP2 or CTSB in the cytoplasm of AGS and its PGC transfectants (Fig. 7B).

## Discussion

PGC expression is markedly upregulated in various cancers, including prostate, breast, endometrial, ovarian, pancreatic, renal, and bladder cancer and eyelid basal cell carcinoma, melanoma, and squamous cell carcinoma, whereas it is either down-regulated or the serum pepsinogen II level is lower in gastric cancer than in healthy controls, which is due to the invasion of large tumors into the stomach body with high numbers of chief cells [3, 10–12]. PGC can break up sperm proteins to ameliorate the vaginal immune load and loosen the extracellular matrix [13] or separate the intercellular connections of cancer cells to form isolated cancer cells for tumor invasion and metastasis [3, 14]. Chen et al. [15] found that PGC expression was inversely associated with a larger tumor size and incomplete encapsulation and can be considered an independent prognostic factor for long overall and disease-free survival in hepatocellular carcinoma. Díaz et al. [16] found that PGC could be used to predict a longer overall survival and indicate androgen dependency in prostate cancer. Immunohistochemically, PGC expression is significantly higher in well-differentiated than in moderately and poorly- differentiated adenocarcinoma of the pancreas, breast, and stomach [17–19]. Fernandez et al. [18] found a negative correlation of PGC expression with lymph node metastasis and poor prognosis of both overall and resectable gastric cancer as an independent predictor of outcome. In the present study, we found that PGC expression was negatively linked to aggressive features, including dedifferentiation, depth of invasion, lymph node metastasis, and short survival of gastric cancer, indicating that PGC loss might be a useful marker of progression and a favorable prognosis. In addition, PGC expression was positively linked to the ER and PR expression of breast cancer and was lowest in TNBC, suggesting that PGC loss is closely linked to the tumorigenesis and histogenesis of TNBC.

To investigate the biological functions of PGC, we for the first time overexpressed PGC in AGS cells and silenced it in GES-1 cells. PGC expression was found to suppress the proliferation, anti-apoptosis, migration and invasion of gastric cancer cells, but versa for PGC knockdown in gastric epithelial cells. These findings suggested that PGC might be considered as a potential target for gene therapy of gastric cancer. According to bioinformatics prediction, our knowledge, Co-IP and double staining, we found that PGC bound to CCNT1, CNDP2 and CTSB. Reportedly, CCNT1 is related to T lymphocyte differentiation and malignant transformation by interacting with CDK9 [20]; CNDP2 is a nonspecific metallopeptidase for the hydrolysis of carnosine and several other dipeptides [21]; CTSB is a lysosomal cysteine endopeptidase and associated with metastasis of cancer cells [22]. Therefore, we speculated that the partner proteins of PGC might be involved in the regulation of aggressiveness of gastric cancer cells, such as proliferation and metastasis.

To verify the relationship between PGC expression and gastric carcinogenesis, we established PGC KO mice and found no difference in body weight and length, serum amino acid level and histological appearance of gastric mucosa between WT and PGC KO mice. Subsequently, we orally administered MNU, a gastric carcinogen, to these mice and discovered protective effects of PGC on chemically-induced gastric carcinogenesis, which might be attributable to a lack of a PGC injury in the gastric epithelium of these KO mice. Reportedly, pepsinogen was decreased at both mRNA and protein levels in the pyloric mucosa of rats treated with another gastric carcinogen, MNNG [23–25], suggesting that chemical carcinogen could lead to dysfunction in chief cells. Tatematsu et al. [26] demonstrated that the susceptibility of rats to MNNG-induced gastric cancer was closely linked to pepsinogen 1-decreased pyloric glands, indicating that these glands represented preneoplastic lesions in chemically-induced gastric carcinogenesis. Taking these findings together, we conclude that PGC loss is the result of gastric carcinogenesis and acts as a protective factor for chemical induction of gastric cancer.

Distinct from PGC-negative gastric cancer, gastric adenocarcinoma of the fundic gland type (GA-FG-CCP) is a rare variant of a well-differentiated and chief cell-predominant adenocarcinoma that is distributed to the fundus and cardia and characterized by frequent submucosal invasion, rare lymphatic and venous invasion, low-grade malignancy, low Ki-67 expression, high PGC and RUNX3 immunopositivity, nuclear β-catenin accumulation, and mutation or hypomethylation of the CTNNB1 or AXIN gene [27–32]. Gastric chief cells are reported to originate from mucous neck cells and to mature into oxyntic glands with secretory zymogen granules. After the loss of parietal cells, chief cells transdifferentiate into mucous cell metaplasia, which is called spasmolytic polypeptide-expressing metaplasia (SPEM) [33]. Meyer et al. [34] demonstrated that damage to the gastric epithelium initiated the conversion of zymogenic chief cells into SPEM, which requires xCT-dependent reactive oxygen species. To investigate the role of chief cells in gastric carcinogenesis, we developed PGC-cre transgenic mice and found that cre activity was not only observed in the gastric, intestinal, and alveolar epithelium, but also, for the first time, in the renal tubule, as evidenced by cre expression, and PTEN deletion. PGC-cre mouse might be useful as a tool to explore the effects of genetic alterations in PGC-positive cells, particularly chief cells.

Here, we found that PTEN abrogation using PGC-cre resulted in gastric cancer and triple-negative breast lobular carcinogenesis. Breast cancer was previously observed in K19-cre-mediated conditional PTEN KO mice as well [8]. Annunziato et al. [35] intraductally injected lentiviral vectors encoding cre and a CRISPR/Cas9 system targeting PTEN into female mice carrying the CDH1 (E-cadherin) ^f/f^ mutation and found the development of invasive lobular breast carcinoma. Li et al. [36] generated PTEN^f/f^/MMTV-cre mice, which exhibited premature lobular development, exorbitant ductal branching, delayed growth, and significantly decreased apoptosis and ultimately developed breast tumors early in life. Schade et al. [37] used MMTV promoter to couple ErbB-2 and cre expression to mammary epithelial cells (MMTV-NIC) and established PTEN-deficient/NIC mice, which showed dramatically accelerated formation of multifocal and highly metastatic breast tumors. The same breast cancer model was generated by crossing transgenic ErbB-2(KI)-ErbB-2 mice with PTEN^f/f^ and MMTV-cre mice [38]. Taken together, unique target PTEN abrogation in PGC-positive cells was strong enough to trigger the development of triple-negative breast lobular adenocarcinoma, which provides a tool to investigate the molecular mechanisms and treatment of TNBC.

Germline mutation, large deletions, and hypermethylation of CDH1 occurring before 40 years of age are also closely linked to hereditary diffuse gastric cancer. It is grossly of no distinct mass and a thick wall of the stomach (linitis plastica) and histologically an autosomal dominant diffuse-type carcinoma, including signet ring carcinoma or isolated cell-type carcinoma [39, 40]. Humar et al. [41] administered MNU to CDH1 ( ±) mice and induced gastric signet ring cell carcinoma with hypoproliferation, low nuclear β-catenin accumulation, and weak membrane E-cadherin expression. Shimada et al. [7] generated an E-cadherin/p53 double conditional KO mouse model using Atp-4b-cre model mice and observed murine signet ring cell carcinoma. Notably, hereditary diffuse gastric cancer has a high risk of lobular breast adenocarcinoma in women. Here, we deleted PTEN from gastric chief cells but found lobular TNBC in PGC-cre/PTEN^f/f^ mice. Therefore, we should pay attention to breast carcinogenesis in patients with hereditary gastric cancer history.

In this study, breast cancer was observed in PGC-cre/PTEN^f/f^ mice with two or three previous pregnancies. There was no breast cancer in virgin PGC-cre/PTEN^f/f^ mice with or without estrogen or progesterone treatment or in PGC-cre/PTEN^f/f^ mice that were exposed to estrogen after one or two previous deliveries. Coughlin et al. [42] determined that a younger age at menarche and an older age at first pregnancy increased breast cancer risk due to long-term exposure to a high estrogen level. We also analyzed the relationship between pregnancy and breast carcinogenesis in women and found that pregnancy decreased the risk of breast cancer, in contrast to hereditary and spontaneous TNBC, which is consistent with the opinion that breastfeeding represents a protective mechanism only in patients without genetic breast cancer-predisposing mutations [43]. Therefore, the results suggest that a reduction in either parity number or breast feeding could help to prevent hereditary breast cancer.

In conclusion, downregulation or loss of PGC expression might favor the development of gastric carcinogenesis. PGC expression suppressed the proliferation, anti-apoptosis, migration and invasion of gastric cancer cells possibly by interaction with CCNT1, CNDP2 and CTSB. Its deletion only results in resistance to chemically-induced gastric carcinogenesis. Spontaneous gastric cancer and triple-negative lobular adenocarcinoma were observed in PGC-cre/PTEN^f/f^ mice. The hereditary breast carcinogenesis might be closely linked to either pregnancy and breast feeding, but not to single exposure to estrogen or progesterone. Therefore, for hereditary breast cancer, fertility and breastfeeding can be considered, or the number of births and breastfeeding can be controlled.

## Supplementary Information

**Additional file 1.**

**Additional file 2.**

## Data Availability

The datasets used and/or analyzed are available from the corresponding author on reasonable request.

## Abbreviations

PGC: Pepsinogen C
WT: Wild-type
KO: Knockout
Atp4b: β-Subunit of H ( +)-, K( +)-ATPase gene
MNU: N-nitroso-N-methylurea
SPF: Specific pathogen-free
ER: Estrogen receptor
PR: Progesterone receptor
PKO: PGC knockout
TNBC: Triple-negative breast cancer
GA-FG-CCP: Gastric adenocarcinoma of the fundic gland type
SPEM: Spasmolytic polypeptide-expressing metaplasia
MMTV: Mouse mammary tumor virus
MNNG: N-methyl-N'-nitro-N-nitrosoguanidine
FBS: Fetal bovine serum
FITC: Fluorescein isothiocyanate isomer
PE: Phycoerythrin
WT: Wild-type
Co-IP: Co-immunoprecipitation
KO: Knock-out
ATP4B: ATPase H + /K + transporting subunit β
CCNT1: Cyclin T1
CDK9: Cyclin-dependent kinase 9
ESR1: Estrogen receptor 1
DHH: desert hedgehog protein
MAT2B: Methionine adenosyltransferase 2 subunit β
TEX101: Testis-expressed protein 101
WFDC8: WAP four-disulfide core domain protein 8
CTSB: Cathepsin B
CNDP2: Carnosine dipeptidase 2
F13B: Coagulation factor XIII B chain
PGM1: Phosphoglucomutase 1
AK2: Adenylate kinase 2
MPG: N-Methylpurine DNA glycosylase
VPS18: Vacuolar protein sorting 18
PDE5A: Phosphodiesterase 5A
NPR1: Natriuretic peptide receptor 1)
APOA1: Apolipoprotein A1
FGG: Fibrinogen γ chain
PTEN: Phosphatase and tensin homolog
