# Supplementary material for: The relationship between pepsinogen C and gastric carcinogenesis: a transgene and population study
Source: BMC Cancer. 2023 Jun 8;23:520. doi: 10.1186/s12885-023-11020-z (PMC10249301; doi:10.1186/s12885-023-11020-z)
Supplement: Supplementary file 1 — Additional file 1. [file 12885_2023_11020_MOESM1_ESM.docx]

**Supplementary table 1.** The spontaneous tumorigenesis in PGC-cre+/-;PTEN-/- mice

| Number | Gender | Month | Cancer types |
| --- | --- | --- | --- |
| 1 | ♀ | 10.5 | breast cancer |
| 2 | ♀ | 11 | breast cancer |
| 3 | ♀ | 12 | breast cancer |
| 4 | ♀ | 11 | breast cancer |
| 5 | ♀ | 10.5 | breast cancer |
| 6 | ♀ | 11 | breast cancer |
| 7 | ♀ | 11.5 | breast cancer |
| 8 | ♀ | 12.5 | breast cancer |
| 9 | ♀ | 12 | breast cancer |
| 10 | ♀ | 11.5 | breast cancer |
| 11 | ♀ | 12.5 | breast cancer |
| 12 | ♀ | - | - |
| 13 | ♀ | 11.5 | breast cancer |
| 14 | ♀ | 12.5 | breast cancer |
| 15 | ♀ | 13.5 | breast cancer |
| 16 | ♂ | - | - |
| 17 | ♂ | - | - |
| 18 | ♂ | - | - |
| 19 | ♂ | 14 | gastric cancer |
| 20 | ♂ | - | - |
| 21 | ♂ | - | - |
| 22 | ♂ | - | - |
| 23 | ♂ | 14 | gastric cancer |

**Supplementary table 2.**The relationship between pregnancy and breast carcinogenesis

| **Pregnancy**  **(times)** | **n** |  | **TNBC** | | **Luminal-type** | | | **Her-2 positive** | | |  |  |  |  |
| --- | --- | --- | --- | --- | --- | --- | --- | --- | --- | --- | --- | --- | --- | --- |
|  |  | **n(%)** | **DC** | **LC** | **n** | **DC** | **LC** | **n** | **DC** | **LC** |  |  |  |  |
| 0 | **85(2.7)** | 13(2.4) | 12 | 1 | 40(1.8) | 40 | 0 | 32(7.0) | 32 | 0 |  |  |  |  |
| 1 | **1312(41.4)** | 217(41.2) | 211 | 6 | 917(42.0) | 844 | 73 | 178(38.9) | 173 | 5 |  |  |  |  |
| 2 | **934(29.5)** | 156(29.6) | 151 | 5 | 644(29.5) | 595 | 49 | 134(29.3) | 133 | 1 |  |  |  |  |
| 3 | **485(15.3)** | 85(16.1) | 81 | 4 | 331(15.2) | 297 | 34 | 69(15.1) | 67 | 2 |  |  |  |  |
| >3 | **350(11.1)** | 56(10.6) | 53 | 3 | 249(11.4) | 228 | 21 | 45(9.8) | 45 | 0 |  |  |  |  |
| Total | **3166** | 527 | 508 | 19 | 2181 | 2004 | 177 | 458 | 450 | 8 |  |  |  |  |
| Note: TNBC, triple-negative breast cancer; DA, ductal carcinoma; LA, lobular carcinoma. | | | | | | | | | | | | |  |  |
