# Supplementary material for: The relationship between pepsinogen C and gastric carcinogenesis: a transgene and population study
Source: BMC Cancer. 2023 Jun 8;23:520. doi: 10.1186/s12885-023-11020-z (PMC10249301; doi:10.1186/s12885-023-11020-z)
Supplement: Supplementary file 2 — Additional file 2. [file 12885_2023_11020_MOESM2_ESM.pptx]

## Slide 1
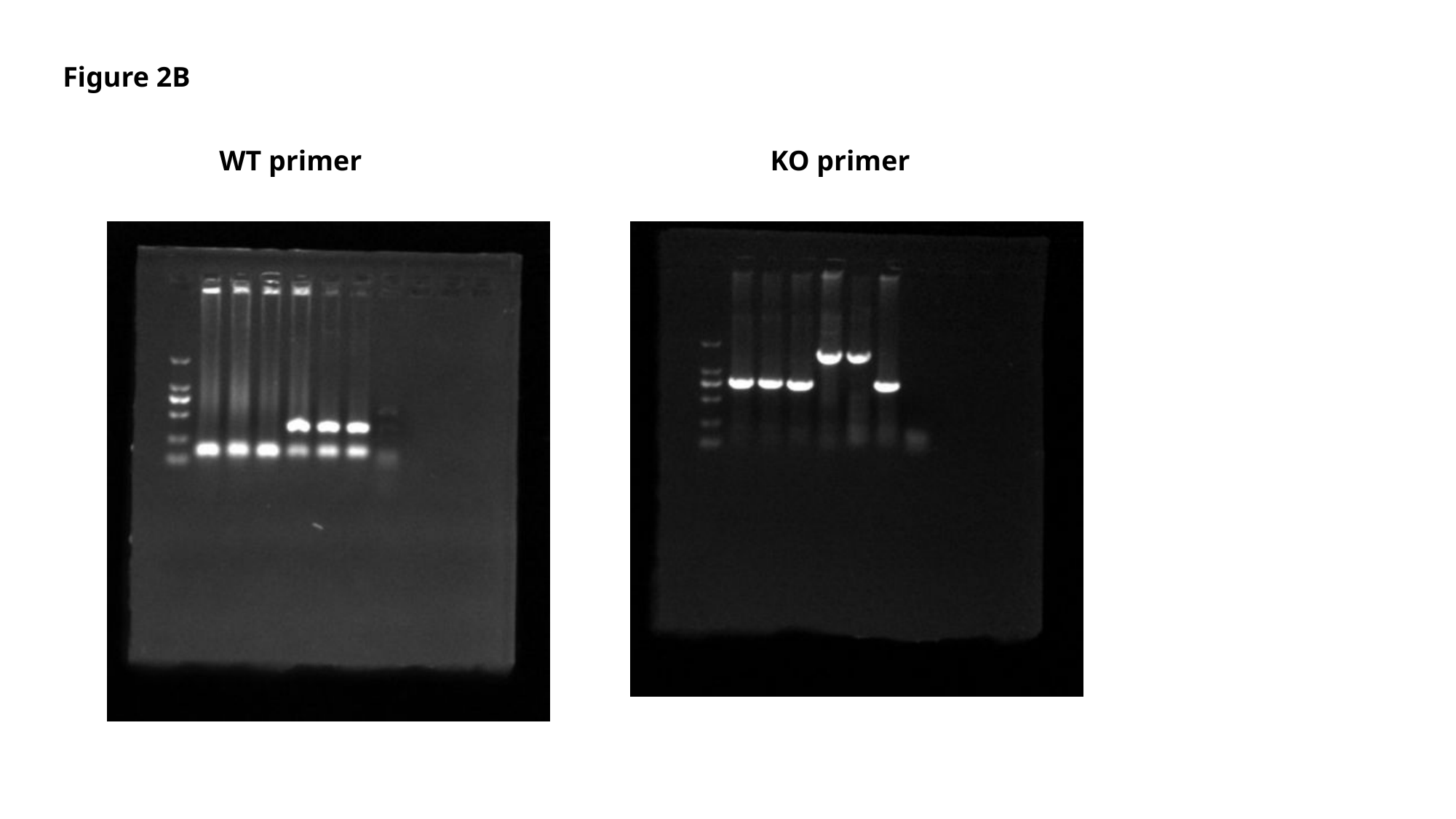

Figure 2B
WT primer
KO primer

## Slide 2
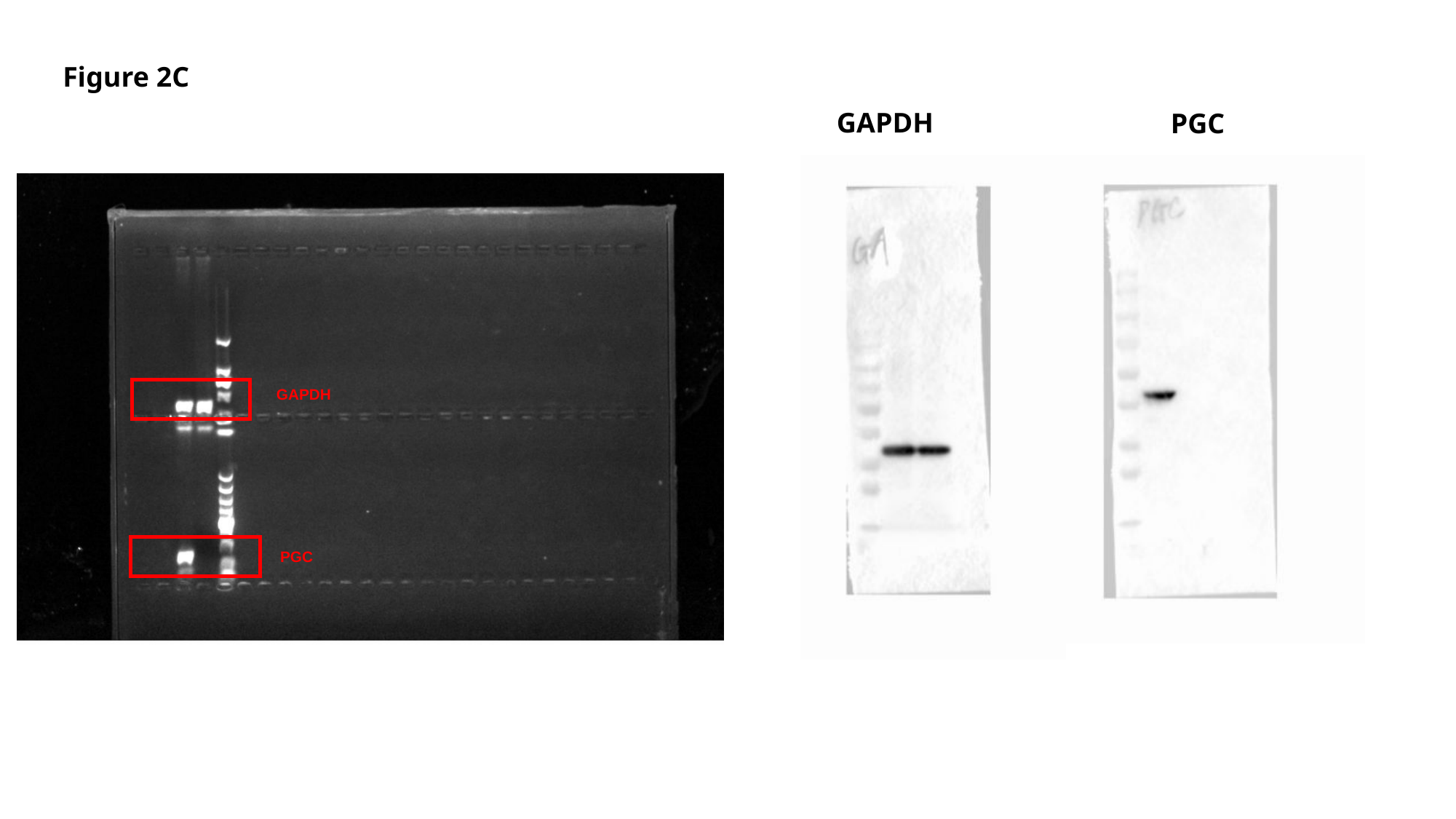

Figure 2C
GAPDH
PGC
GAPDH
PGC

## Slide 3
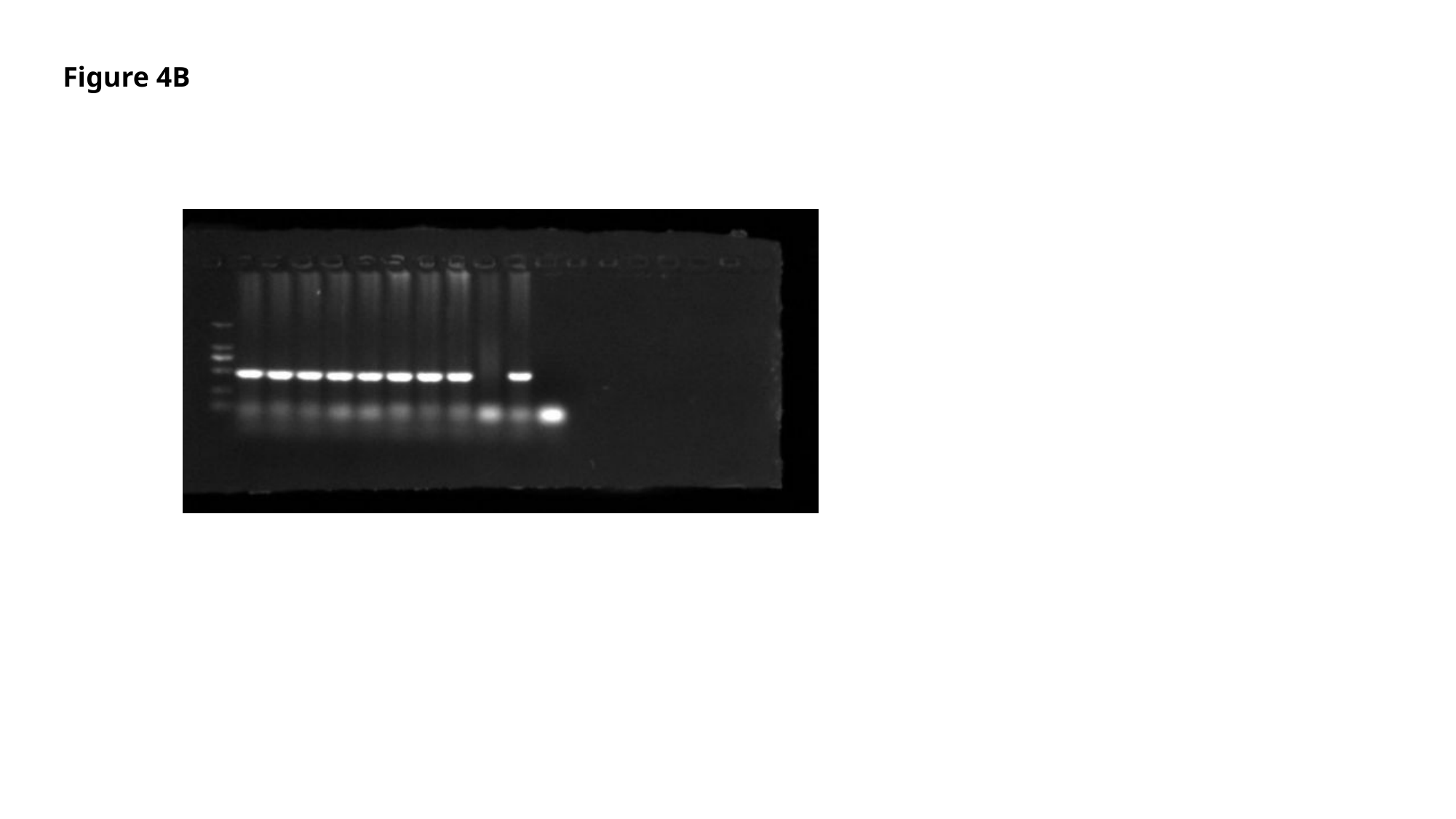

Figure 4B

## Slide 4
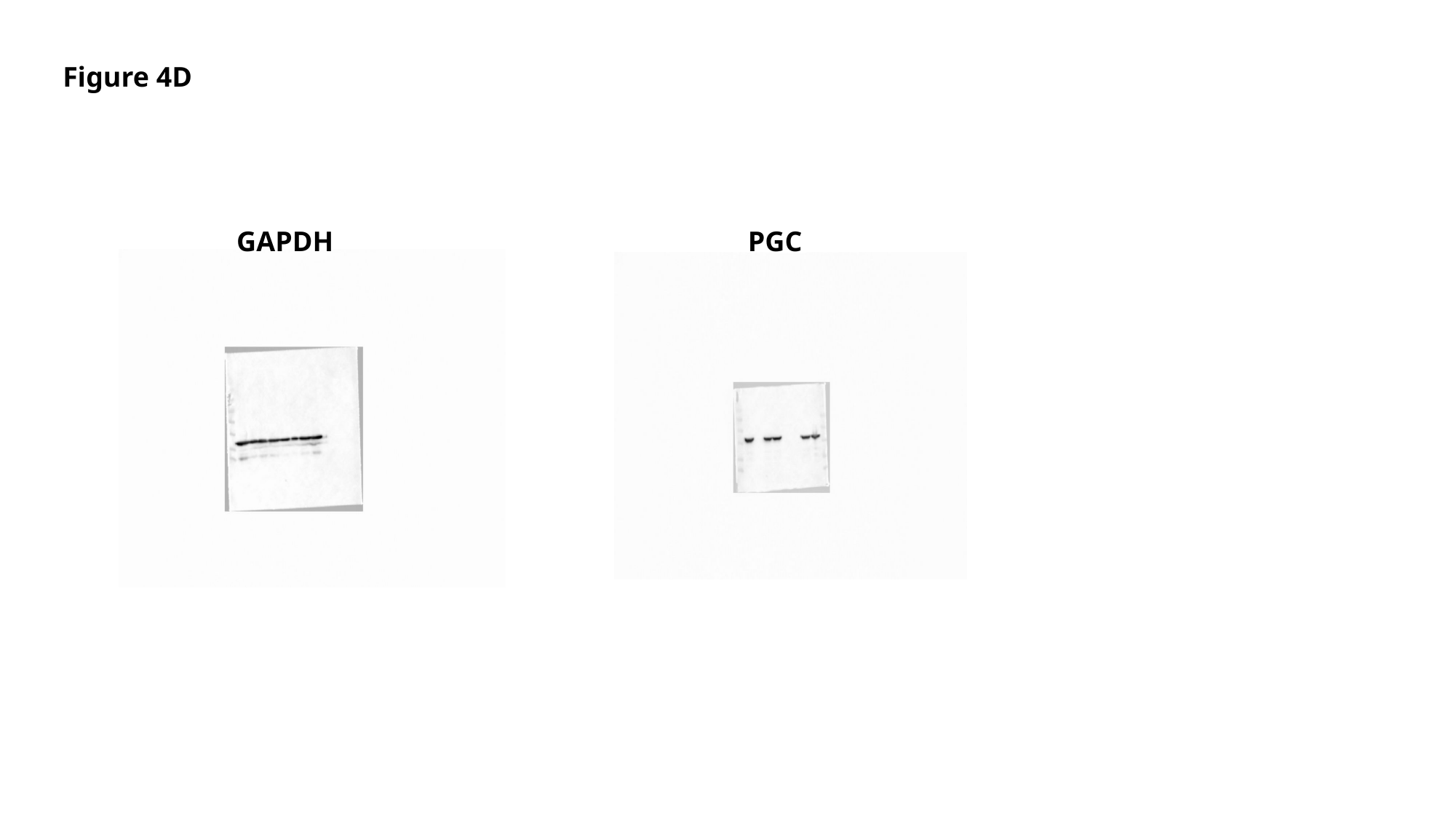

Figure 4D
GAPDH
PGC

## Slide 5
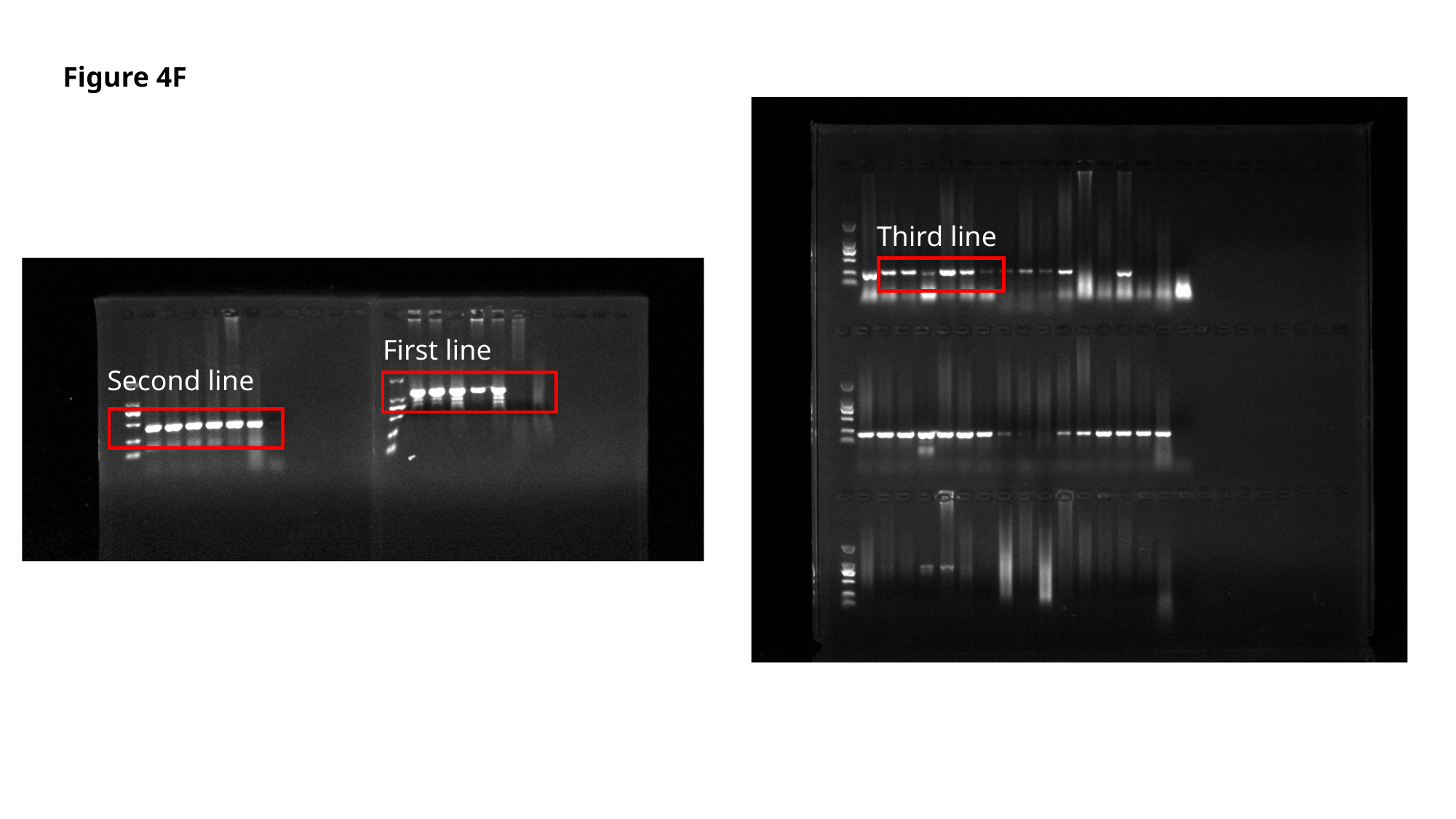

Figure 4F
Third line
First line
Second line

## Slide 6
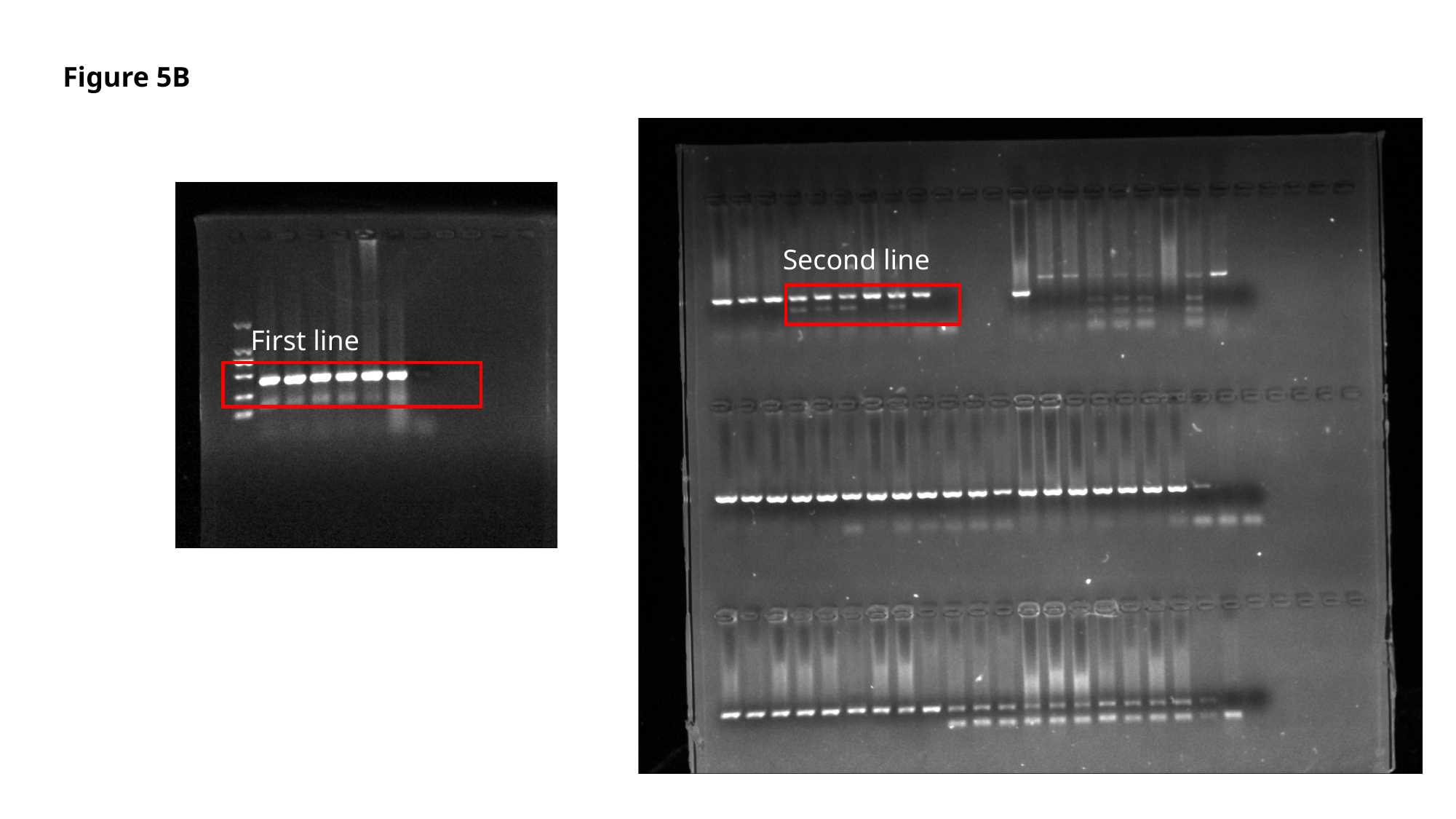

Figure 5B
Second line
First line

## Slide 7
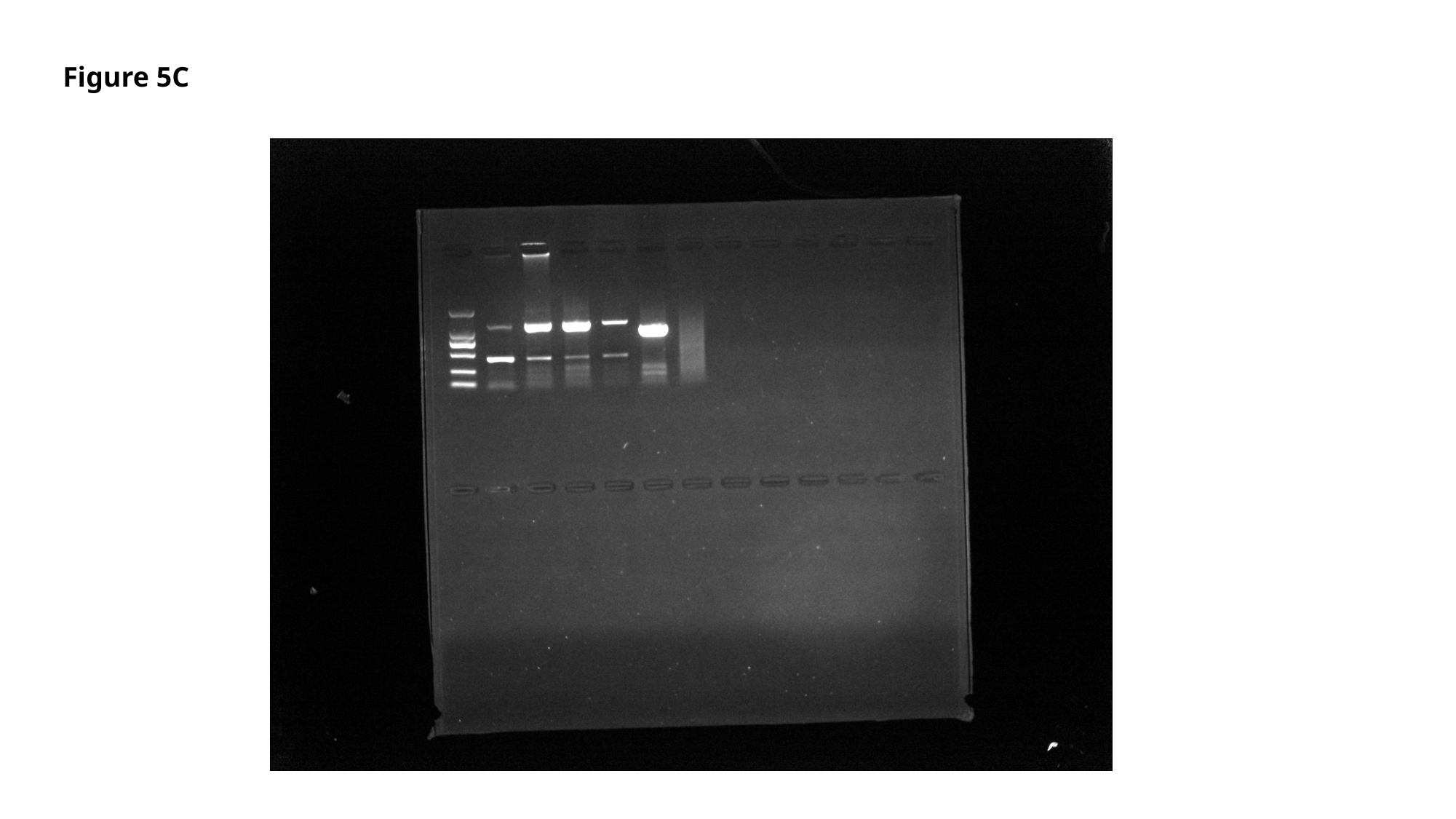

Figure 5C

## Slide 8
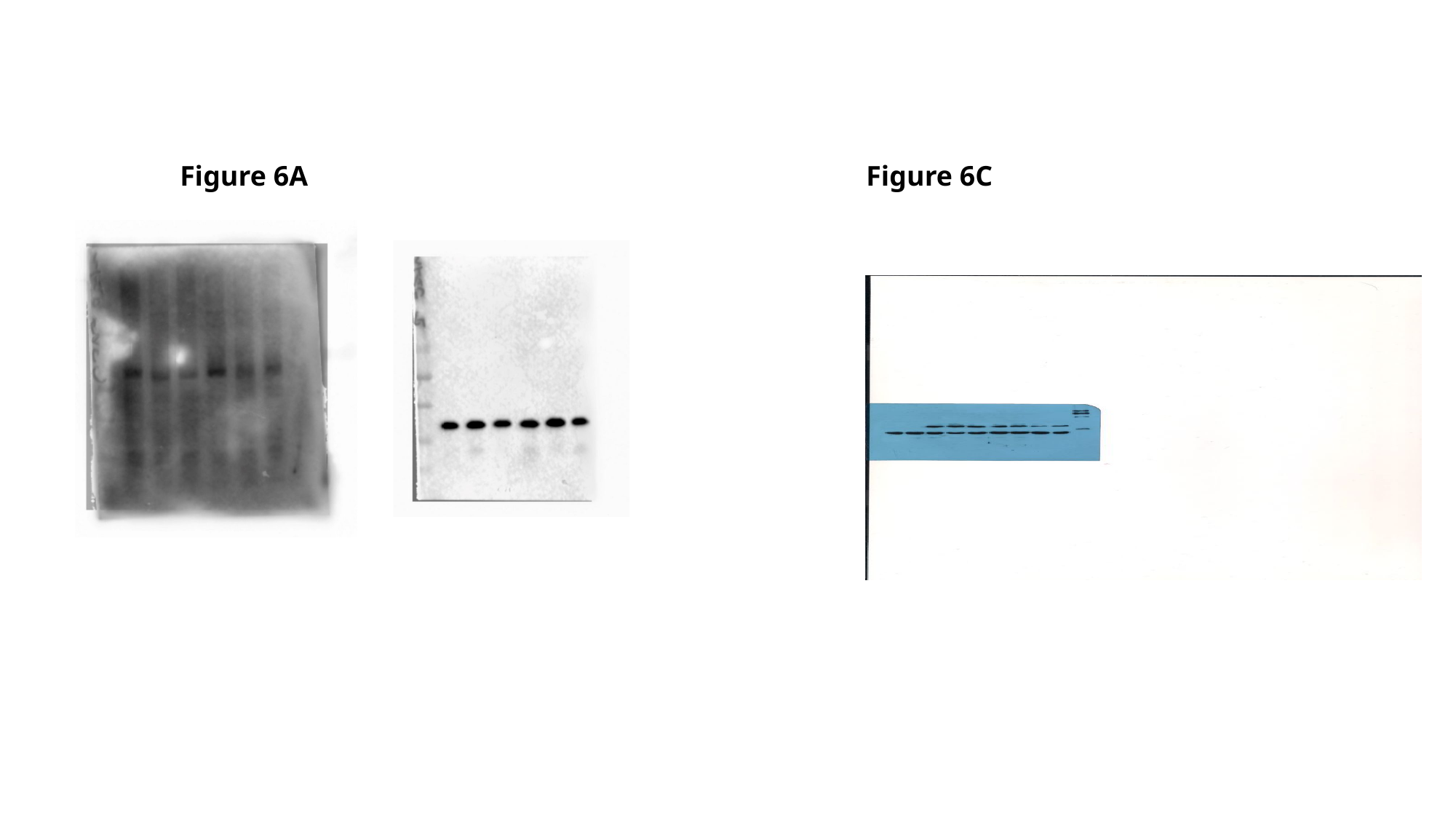

Figure 6A
Figure 6C

## Slide 9
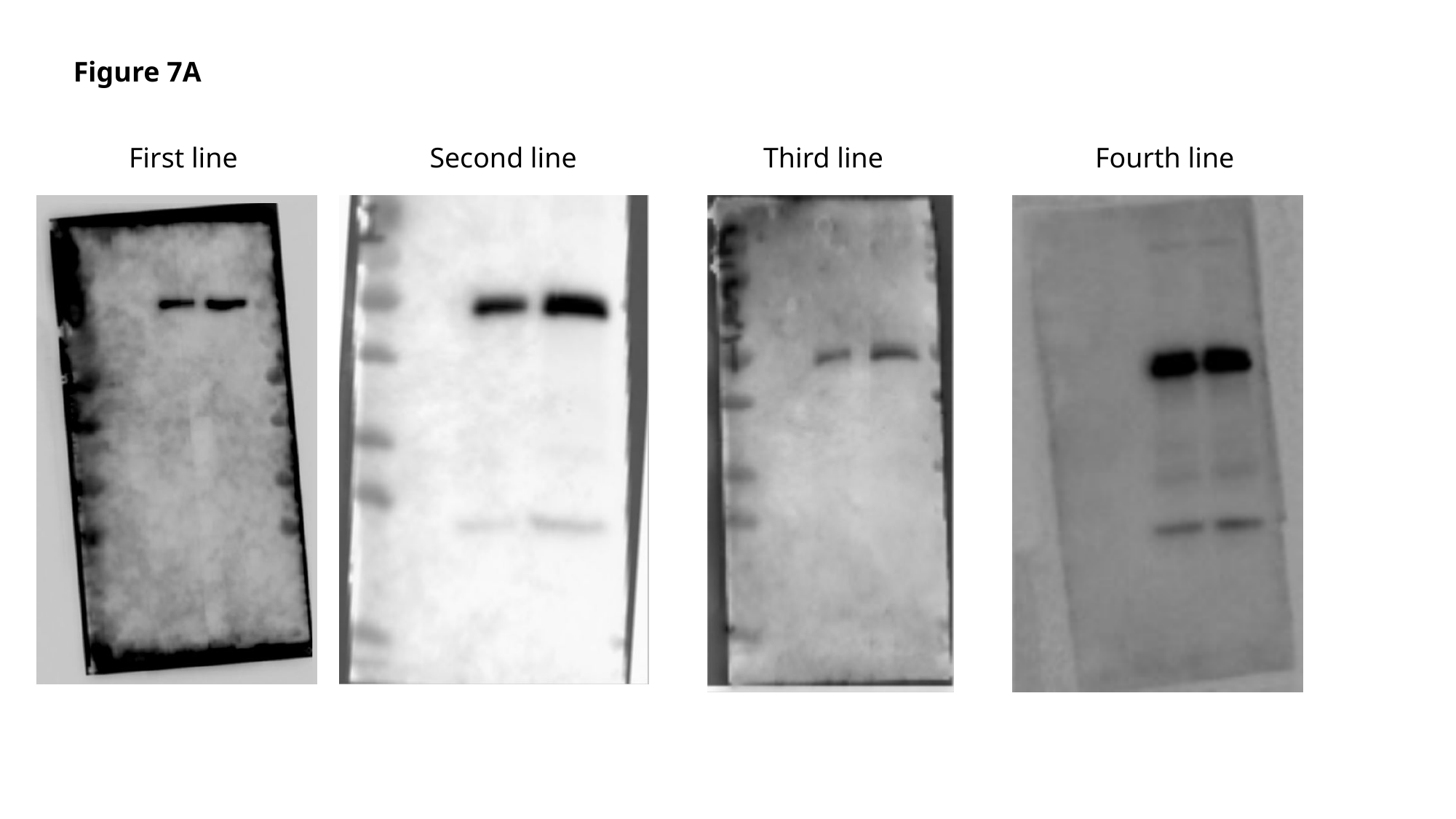

Figure 7A
First line
Second line
Third line
Fourth line

## Slide 10
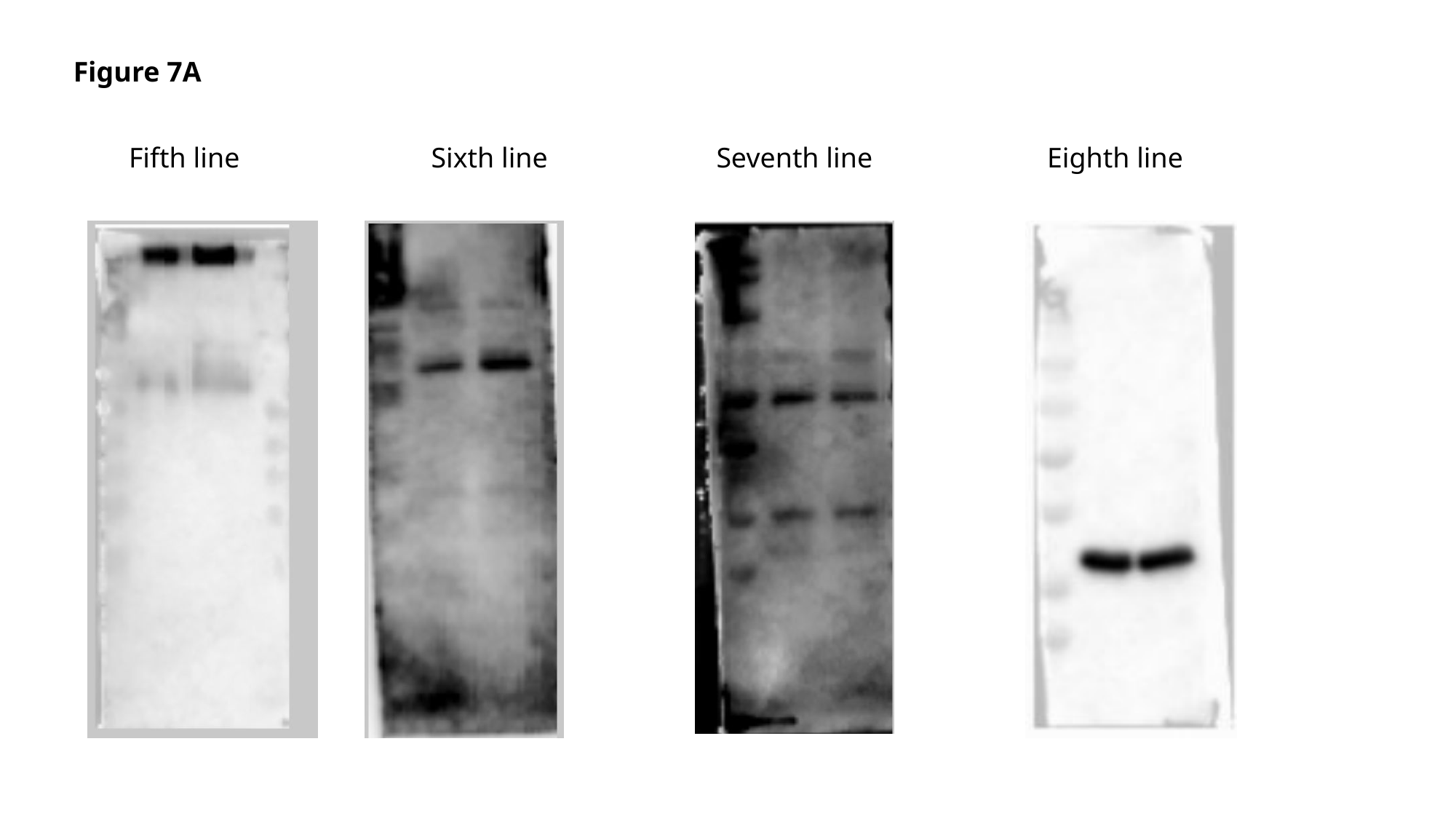

Figure 7A
Fifth line
Sixth line
Seventh line
Eighth line
